# Clinical characteristics and organ system involvement in sarcoidosis: comparison of the University of Minnesota Cohort with other cohorts

**DOI:** 10.1186/s12890-020-01191-x

**Published:** 2020-06-01

**Authors:** Hok Sreng Te, David M. Perlman, Chetan Shenoy, Daniel J. Steinberger, Rebecca J. Cogswell, Henri Roukoz, Erik J. Peterson, Lin Zhang, Tadashi L. Allen, Maneesh Bhargava

**Affiliations:** 1 Division of Pulmonary, Allergy, Critical Care and Sleep Medicine, Department of Medicine, Univesity of Minnesota Medical School, Minneapolis, USA; 2 Cardivascular Division, Department of Medicine, Univesity of Minnesota Medical School, Minneapolis, USA; 3 Division of Rheumatic and Autoimmune Diseases, Department of Medicine, University of Minnesota Medical School, Minneapolis, MN USA; 4 Department of Radiology, University of Minnesota Medical School, Minneapolis, USA; 5 Division of Biostatistics, School of Public Health, University of Minnesota, Minneapolis, MN USA

**Keywords:** Sarcoidosis, Cohort, Clinical characteristics, Organ system involvement, Clinical phenotyping

## Abstract

**Background:**

Sarcoidosis is a systemic granulomatous disease of unknown etiology. Clinical cohort studies of different populations are important to understand the high variability in clinical presentation and disease course of sarcoidosis. The aim of the study is to evaluate clinical characteristics, including organ involvement, pulmonary function tests, and laboratory parameters, in a sarcoidosis cohort at the University of Minnesota. We compare the organ system involvement of this cohort with other available cohorts.

**Methods:**

We conducted a retrospective data collection and analysis of 187 subjects with biopsy-proven sarcoidosis seen at a tertiary center. Organ system involvement was determined using the WASOG sarcoidosis organ assessment instrument. Clinical phenotype groups were classified using the Genomic Research in Alpha-1 Antitrypsin Deficiency and Sarcoidosis criteria.

**Results:**

Mean subject age at diagnosis was 45.8 ± 12.4, with a higher proportion of males (55.1%), and a higher proportion of blacks (17.1%) compared to the racial distribution of Minnesota residents (5.95%). The majority (71.1%) of subjects required anti-inflammatory therapy for at least 1 month. Compared to the A Case Control Etiologic Study of Sarcoidosis cohort, there was a higher frequency of extra-thoracic lymph node (34.2% vs. 15.2%), eye (20.9% vs. 11.8%), liver (17.6% vs. 11.5%), spleen (20.9% vs. 6.7%), musculoskeletal (9.6% vs. 0.5%), and cardiac (10.7% vs. 2.3%) involvement in our cohort. A multisystem disease with at least five different organs involved was identified in 13.4% of subjects. A restrictive physiological pattern was observed in 21.6% of subjects, followed by an obstructive pattern in 17.3% and mixed obstructive and restrictive pattern in 2.2%. Almost half (49.2%) were Scadding stages II/III. Commonly employed disease activity markers, including soluble interleukin-2 receptor and angiotensin-converting enzyme, did not differ between treated and untreated groups.

**Conclusions:**

This cohort features a relatively high frequency of high-risk sarcoidosis phenotypes including cardiac and multiorgan disease. Commonly-utilized serum biomarkers do not identify subpopulations that require or do better with treatment. Findings from this study further highlight the high-variability nature of sarcoidosis and the need for a more reliable biomarker to predict and measure disease severity and outcomes for better clinical management of sarcoidosis patients.

## Background

Sarcoidosis is a multi-system disease of unknown etiology characterized by the presence of non-caseating granulomas in involved organs [1]. Although lungs, mediastinal and hilar lymph nodes are most commonly involved, sarcoidosis is associated with heterogeneous manifestations, disease severity, and outcomes [1–4]. The incidence of sarcoidosis is at least 45–300/100,000 individuals in the US [5], of both sexes, and all ages and races [1, 6–8]. The annual mortality is 2.8/million people [3, 9] and is rising [10] with a more aggressive course and more extrapulmonary involvement reported in black patients [11].

Recently, four high-risk sarcoidosis manifestations were recognized as highly associated with greater morbidity and mortality [12]. These include treatment-resistant pulmonary disease, neuro-sarcoidosis, cardiac sarcoidosis, and multisystem organ involvement. In the US and Europe, respiratory failure is the most common cause of sarcoidosis-related mortality [9, 10]. In contrast, Japanese cohorts have a higher incidence of cardiac disease, and cardiac involvement is the most frequent case of death due to sarcoidosis in Japan [13]. Lofgren’s syndrome, an acute presentation of sarcoidosis associated with fever, erythema nodosum, arthralgias and mediastinal or hilar adenopathy, is more frequent in Scandinavian countries and is associated with a good prognosis [14]. The heterogeneity in sarcoidosis presents challenges in predicting disease course, optimizing treatments, and establishing a uniform standard for organ involvement assessment.

There is significant geographic variability in sarcoidosis manifestations. The variability in sarcoidosis manifestations could be explained by regional variability in sarcoidosis but also could be due to the tools employed for assessment of organ involvement. A number of reports have used the tool developed by the A Case Control Etiologic Study of Sarcoidosis (ACCESS) investigators [15] to assess disease manifestation [11, 16, 17]. This tool was further modified by the World Association of Sarcoidosis and Other Granulomatous Disorders (WASOG) task force to address additional manifestations and possible organs involved with sarcoidosis. The WASOG sarcoidosis organ assessment instrument classifies organ involvement as “highly probable”, “probable”, “possible”, or “no consensus” [18]. Various other assessment tools for sarcoidosis have been proposed. More recently, the Genomic Research in Alpha-1 Antitrypsin Deficiency and Sarcoidosis (GRADS) study classified clinical phenotypes into nine clinical phenotype groups [19], including efforts to define multiorgan, cardiac defining therapy, and other phenotypes.

Our objective is to describe the clinical characteristics of the sarcoidosis cases seen at the University of Minnesota, and to compare the characteristics of our cohort to the ACCESS and other cohorts. The racial distribution of Minnesota residents includes White (83.75%), Black or African American (5.95%), Asian (4.66%), two or more races (2.81%), other race (1.74%), Native American (1.05%), and Native Hawaiian or Pacific Islander (0.04%) [20]. A unique feature of the Minnesota population is the substantially higher percentage of residents of Scandinavian origin which may influence the disease manifestations. While only 0.2% of US population is of Scandinavian origin, up to 1.5% of Minnesota residents self-report a Scandinavian origin, and we presume that our cohort may have this representation [21].

## Methods

This cohort includes 187 sarcoidosis subjects consented between March 2015 and May 2019 at a tertiary sarcoidosis referral center in Minnesota. The diagnosis of sarcoidosis was made per American Thoracic Society (ATS) statement [1], meeting the following criteria: 1) a compatible clinical and radiologic finding; 2) histological evidence of non-caseating granulomas; and 3) exclusion of other diseases such as infections, common variable immunoglobulin deficiency (CVID), chronic beryllium disease (CBD), and malignancy. Diagnosis dates were determined per biopsy dates and estimated to be June 15th when days and/or months were not available. Subjects without histological evidence were excluded from this cohort. Subjects with a history of malignancy diagnosed within two years prior to or after sarcoidosis were excluded to exclude the possibility of sarcoid-like reactions to malignancies.

We conducted a chart review to collect data on demographics, diagnostics, imaging, past medical history, treatment program, and pulmonary function tests. Racial demographics were collected per patient self-report through the electronic medical record. With limited data, Hispanic whites and non-Hispanic whites were both categorized as “White” in this study. Results from laboratory within one year prior to the initial visit were also collected, including angiotensin converting enzyme (ACE), white blood count (WBC) with differential, soluble interleukin-2 receptor (sIL-2R), C-reactive protein (CRP), immunoglobulin G (IgG), albumin, total protein, liver function tests, calcium, parathyroid hormone (PTH), 25-OH vitamin D, and 1,25-DiOH vitamin D. Scadding stage was established using the closest available imaging study - chest x-ray (CXR) and chest CT topogram if CXR was not available - by an expert independent chest radiologist. The treatment groups, organ involvement, and clinical phenotype were assessed based on the characteristics at enrollment and prior history.

In the absence of histological evidence, the organ system involvement was determined using the ‘highly probable“ and “probable” criteria per the WASOG sarcoidosis organ assessment instrument [18]. Per the WASOG instrument, thoracic involvement includes both pulmonary involvement and thoracic lymph node involvement. One modification we made from the instrument was that although arthralgia was listed under “possible” criteria, we included it for bone-joint involvement when it was associated with Lofgren’s syndrome. Additionally, small fiber neuropathy with positive skin biopsy findings was included for nervous system involvement. In addition to PET, subjects with abnormal signal intensity on magnetic resonance imaging (MRI) of bone marrow were also included for bone marrow involvement.

We established the clinical phenotype groups using the GRADS criteria [19] with minor modifications. Subjects with Lofgren’s syndrome but treated for > 3 months were nonetheless assigned to Acute Sarcoidosis (Group 7). Untreated or treated subjects who were off treatment for at least one year from the visit date were assigned to the Remitting Disease group (Group 8). Subjects with five or more involved organs including cardiac involvement and on systemic anti-inflammatory therapy without remission (not assigned to Group 8) were assigned to Cardiac Defining Therapy (Group 9) as opposed to Multiorgan Disease (Group 1). We identified unclassifiable cases that did not fit any of the GRADS phenotypes, including Stage 0 treated or untreated and Stage I treated.

We grouped the PFT abnormalities into obstructive, restrictive, or mixed obstructive and restrictive ventilatory defects. Obstructive ventilatory defect was defined using Global Initiative for Chronic Obstructive Lung Disease criteria as forced expiratory volume in 1 s (FEV1) / forced vital capacity (FVC) ratio (FEV1/FVC) < 70% and a reduction of FEV1 [22]. Restriction ventilatory defect was defined as FEV1/FVC ratio ≥ 70% and a reduction in FVC. A mixed obstructive and restrictive pattern was defined as FEV1/FVC ratio < 70%, a decrease in FEV1 and total lung capacity (TLC). To simplify the interpretation, the FEV1, FVC, TLC and diffusing capacity of the lungs for carbon monoxide (DLCO) were considered reduced if they were below 80% of predicted values.

Statistical analysis was conducted using R version 3.6.1. Categorical variables were analyzed using Chi-square test or Fisher’s exact test when expected values are less than five, while continuous variables were conducted using two-sample Student’s t-test and analysis of variance (ANOVA) when more than two groups were compared, with *p* < 0.05 considered statistically significant.

## Results

### Demographics

We included 187 consecutive sarcoidosis subjects with histological evidence of noncaseating granulomas and a clinical presentation consistent with sarcoidosis (Table 1). The majority of the subjects were white (70.6%) or African American (17.1%). The mean age at diagnosis was 45.8 ± 12.4 years, with no significant difference between males (44.7 ± 12.5 years) and females (47.1 ± 12.1 years), and whites (47.1 ± 12.6 years) and blacks (43.3 ± 10.9 years). More than half of the subjects were males (55.1%). The mean body mass index (BMI) was 31.2 ± 7.1 kg/m^2^, with no significant difference between males (31.4 ± 6.4 kg/m^2^) and females (31.0 ± 7.8 kg/m^2^), and Whites (31.6 ± 6.8 kg/m^2^) and Blacks (29.6 ± 6.4 kg/m^2^). The use of anti-inflammatory medications for at least one month for sarcoidosis was present in 71.1% of subjects. The mean age at diagnosis for the treated group (44.2 ± 12.0 years) who received immunosuppression for ≥1 month was lower compared to cases (50.0 ± 12.4 years) that were never treated or treated < 1 month. The treated group also had a higher mean BMI (32.0 ± 7.5 kg/m^2^ vs 29.4 ± 5.7 kg/m^2^, *p* = 0.01). Only a minority of the subjects (9%) reported a family history of sarcoidosis. Thirty eight percent of subjects were former cigarette smokers, and 8% were current smokers.

**Table 1:** Demographic characteristics of the cohort (*n* = 187)

Characteristics	mean ± SD or *n* (%)
Age at enrollment (years)	53.0 ± 12.6
Age at diagnosis (years)	45.8 ± 12.4
Sex	
Male	103 (55.1%)
Female	84 (44.9%)
Race	
White	132 (70.6)
African American	32 (17.1)
Asian	3 (1.6)
American Indian or Alaska Native	1 (0.5)
Choose not to answer	19 (10.2)
Body mass index (kg/m^2^)	31.2 ± 7.1
Smoking status	
Never	101 (54.0)
Former	71 (38.0)
Current	15 (8.0)
Family history of sarcoidosis	16 (9.0)
Use of anti-inflammatory agents	
Treated ≥1 month	133 (71.1)
Treated < 1 month or never	54 (28.9)
Pulmonary function tests	
FEV1 (% predicted)	80.0 ± 23.5
FVC (% predicted)	85.2 ± 21.2
FEV1:FVC ratio	74.4 ± 11.5
TLC (% predicted)	92.7 ± 17.3
DLCO (% predicted)	89.5 ± 26.0

### Organ involvement

Organ involvement was assessed using the WASOG sarcoidosis organ assessment instrument [18]. Thoracic involvement was detected in 184 (98.4%) subjects by histology or imaging studies with chest x-ray (CXR) and/or CT scan, with the majority of the cases (85.9%) having histological evidence of noncaseating granulomas (Table 2). Extra-thoracic involvement was observed in 128 (68.4%) subjects, 59 (31.6%) subjects had thoracic involvement only, and 3 (1.6%) subjects had extra-thoracic involvement only. Bone and joint involvement were more frequent for whites (8.2% vs 0% for blacks, *p* = 0.04). Otherwise, there was no statistically significant association between each of the organ involvement and sex, race, or treatment group.

**Table 2:** Organ involvement and histological evidence indicating subjects with evidence of noncaseating granulomas

Characteristics	Organ Involvement per WASOG Tool		Histological Evidence		
	n/187	%	95% CI	n/total	%
Lung	184	98.4	95.4–99.7	158/184	85.9
Skin	27	14.4	9.7–20.3	22/27	81.5
Liver	33	17.6	12.5–23.9	12/33	36.4
Eye	39	20.9	15.3–27.4	5/39	12.8
Spleen	39	20.9	15.3–27.4	2/39	5.1
Salivary Gland	4	2.1	0.6–5.4	1/4	25.0
ENT	4	2.1	0.6–5.4	3/4	75.0
Calcium-VitD	10	5.3	2.6–9.6	0/10^a^	0.0
Bone-Joint	18^b^	9.6	5.8–14.8	3/18	16.7
Bone Marrow	6	3.2	1.2–6.9	4/6	66.7
Muscle	2	1.1	0.1–3.8	1/2	50.0
Extra-Thoracic Lymph Node	64	34.2	27.5–41.5	18/64	28.1
Kidney	2	1.1	0.1–3.8	2/2	100.0
Nervous System	14	7.5	4.1–12.2	1/14^c^	7.1
Cardiac	20	10.7	6.7–16.0	3/20	15.0
Other Organs	9	4.8	2.2–8.9	6/9	66.7
Testis	3	1.6	0.3–4.6	2/3	66.7
Large intestine	1	0.5	0.0–2.9	1/1	100.0
Stomach	2	1.1	0.1–3.8	2/2	100.0
Thyroid	2	1.1	0.1–3.8	0/2	0.0
Appendix	1	0.5	0.0–2.9	1/1	100.0

^a^10 subjects with evidence of calcium stones by stone analysis for calcium-vitD involvement

^b^4 subjects with arthralgia associated with Lofgren’s syndrome was included for bone-joint involvement

^c^3 subjects with evidence of small fiber neuropathy by skin biopsy for nervous system involvement

Other organs that were commonly involved included extra-thoracic lymph nodes (34.2%), eye (20.9%), and spleen (20.9%). Extra-thoracic adenopathy was detected by imaging in at least two sites, including cervical, supraclavicular, axillary, paraoesophageal, mesenteric, retroperitoneal, portocaval, hepatogastric, paraaortic, porta hepatis, inguinal, or iliac chain node stations; 28.1% of those with adenopathy underwent biopsy that provided evidence of granulomatous lymphadenitis. Among those with ocular disease, uveitis (61.5%) and lacrimal gland enlargement (18.0%) were the primary manifestations. In cases with splenic involvement, splenomegaly was present in 12 (30.8%), and splenic lesions were detected in 28 (71.8%) by imaging studies. In five subjects, splenomegaly was secondary to portal hypertension from non-alcoholic steatohepatitis (NASH), and these cases were not considered to have sarcoidosis splenic involvement as there was an alternative explanation for the splenomegaly. The majority of subjects (67.9%) had more than one organ involved (Table 3). Multiorgan involvement (at least five different organs involved) was identified in 13.4% of subjects. The mean number of organs involved was higher in treated vs. untreated cases in our cohort (2.7 vs 2.1, *p* = 0.004). Otherwise, there was no significant difference in the mean number of organs involved across gender and racial groups.

**Table 3:** Number of organs involved in each subject

Number of organs	Cases %	
	*n*	%
1	60	32.1
2	50	26.7
3	35	18.7
4	17	9.1
5	14	7.5
6	7	3.7
7	2	1.1
8	2	1.1

High-risk sarcoidosis with cardiac or neurologic involvement was observed in 20 (10.7%) and 14 (7.5%) subjects, respectively. Among subjects with cardiac involvement, three had endomyocardial biopsies demonstrating granulomatous inflammation; eight had a history of ventricular tachycardia and/or high-degree atrioventricular (AV) block such as Morbitz type II or complete heart block found in five subjects; ten had left ventricular ejection fraction below 50%. Nine patients received automatic implantable cardioverter defibrillators and two cases had pacemaker implantation. Advanced cardiac imaging was abnormal with suggestion of myocardial involvement in 16 cases. 18F-fluorodeoxyglucose (FDG) positron emission tomography (PET) performed with a cardiac protocol demonstrated abnormal uptake in 13 subjects, while late gadolinium enhancement (LGE) on cardiac MRI was detected in 9 subjects [23]. One subject underwent heart transplant. For nervous system involvement, three subjects had small fiber neuropathy, followed by three pituitary, two meninges, two spinal cord, one cranial nerve, one white matter, one sensory hearing loss, and one cranio-facial with peripheral neuropathy. Two subjects had both cardiac and neurological involvement.

### Pulmonary function tests and Scadding stage

**Fig. 1:**
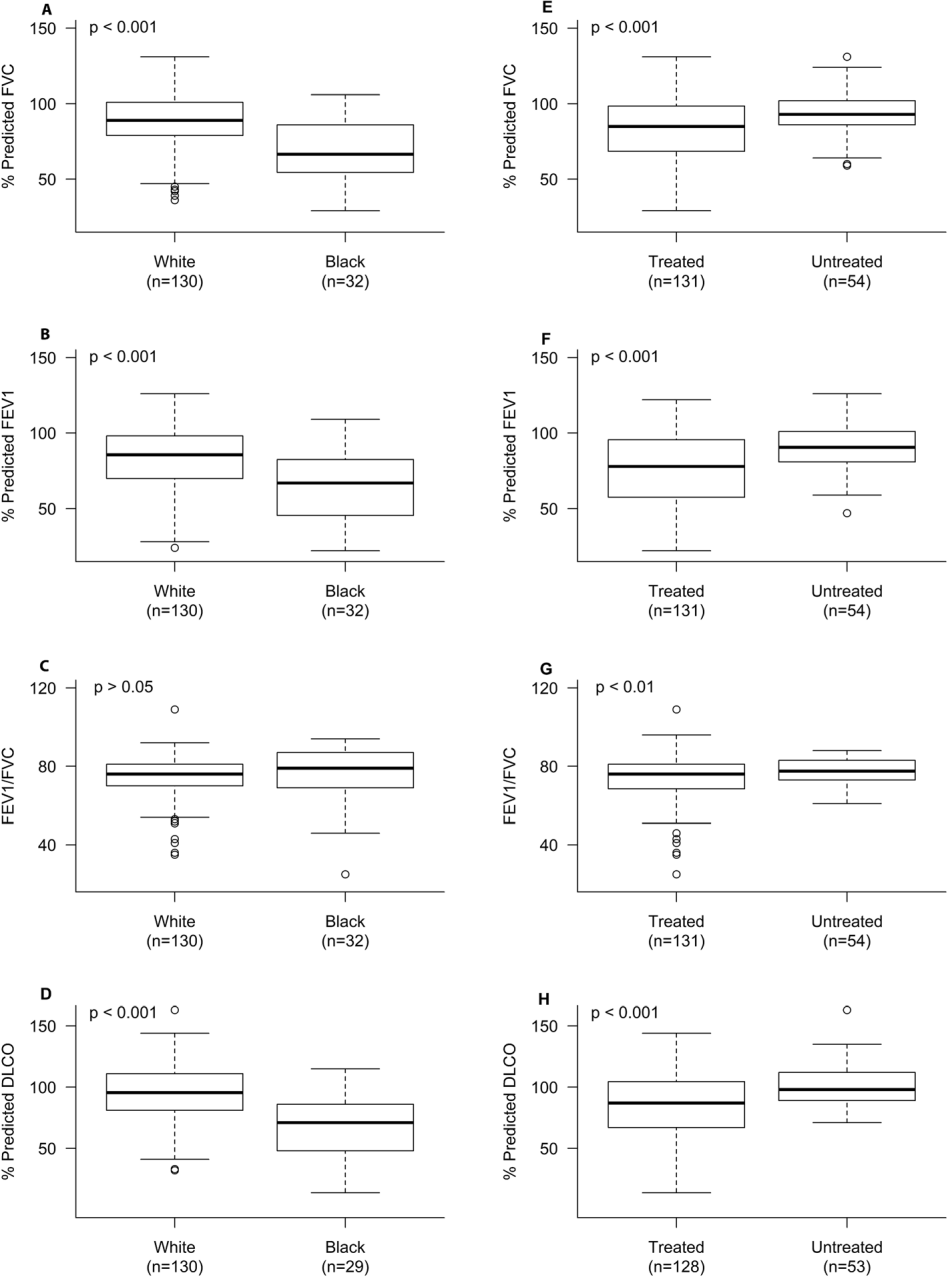
Percent predicted FVC, % predicted FEV1, FEV1/FVC ratio, and % predicted DLCO between treated and untreated groups, and whites and blacks. A statistically significant difference at *p* < 0.001 in the FVC, FEV1, and DLCO was observed between treated and untreated groups, and whites and blacks. For FEV1/FVC ratio, a significant difference at *p* < 0.01 was only observed between treated and untreated groups

Pulmonary function tests were available for 185 subjects. Normal pulmonary function tests were observed in 109 (58.9%) subjects, a restrictive pattern in 40 subjects (21.6%), an obstructive pattern in 32 subjects (17.3%), and mixed obstructive and restrictive pattern in 4 (2.2%) subjects. For FVC, the median and 25th percentile were 88% predicted and 72% predicted, respectively. For FEV1, they were 84% predicted and 67% predicted, and for DLCO, they were 93% predicted and 75% predicted, respectively. Lung volumes were available for 129 subjects, and the median and 25th percentile of TLC were 95% predicted and 82% predicted, respectively. Twenty-nine (22.5%) cases had decreased TLC (below 80% predicted), and 22 (17.1%) had restriction on spirometry and decreased TLC. Decreased TLC with normal spirometry was seen in only 4 (3.1%) cases. Fifty-five (30.4%) subjects had reduced DLCO, while 12 (6.6%) had reduced DLCO with normal spirometry. Eighteen (14.0%) subjects had abnormal DLCO with normal TLC, and 4 (2.2%) had an isolated decrease in DLCO with normal spirometry and lung volumes. Black subjects had lower mean FVC, FEV1, and DCLO than white subjects (*p* < 0.001) (Fig. 1, panel a, b, d). Likewise, subjects treated with anti-inflammatory medications for at least ≥1 month had lower mean FVC, FEV1, and DLCO (*p* < 0.001), and lower FEV1/FVC ratio (*p* = 0.006) compared to untreated subjects (Fig. 1, panel e, f, g, h).

**Fig. 2:**
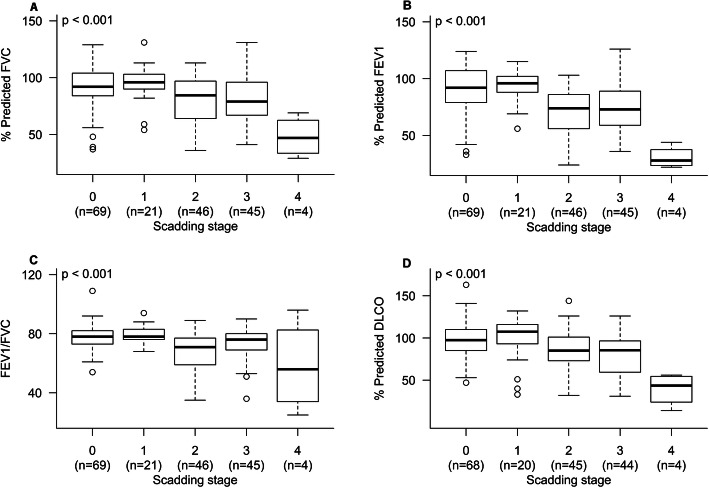
Percent predicted FVC, % predicted FEV1, FEV1/FVC ratio, and % predicted DLCO (not corrected for hemoglobin). The FVC, FEV1, FEV1/FVC ratio, and DLCO were different by Scadding stage, with *p* < 0.001. Further post hoc test with Tukey’s HSD shows significant difference in FVC and FEV1 between Scadding stages 0 and 2, 0 and 3, 0 and 4, 1 and 2, 1 and 3, 1 and 4, 2 and 4, and 3 and 4. For FEV1/FVC ratio, a significant difference was observed between stages 0 and 2, 0 and 4, 1 and 2, 1 and 4, and 3 and 4. Meanwhile, for DLCO, a significant difference was found between stages 0 and 3, 0 and 4, 1 and 4, 2 and 4, and 3 and 4

Ninety-two (49.2%) cases had parenchymal lung involvement with Scadding stage II or III disease. Seventy cases (37.4%) had Scadding stage 0 disease, and 2.1% had primarily fibrotic stage IV (Table 4). Difference in the mean FVC, FEV1, FEV1/FVC and DLCO were observed based on Scadding stage (*p* < 0.001, Fig. 2). The mean FVC was 92 ± 19% for stage 0, 95 ± 17% for stage I, 80 ± 20 for stage II, 79 ± 21 for stage III, and 48 ± 18 for stage IV. The mean FVC was lower in stage IV disease compared to other Scadding stages, suggesting a restrictive pattern in these cases. Cases with parenchymal involvement (stage II or III) had lower mean FVC than those without parenchymal disease (stage 0 or I). The mean DLCO was 97 ± 23% for stage 0, 99 ± 28% for stage I, 86 ± 21% for stage II, 81 ± 26% for stage III, and 39 ± 19% for stage IV. Similar to FVC, the mean DLCO was lower for the Scadding stage IV cases compared to those of other Scadding stages, and although in normal range, the mean DLCO of stage II and II combined was lower than cases with no parenchymal lung disease. The mean FEV1 was 90 ± 20 for stage 0, 94 ± 13 for stage I, 69 ± 21 for stage II, 74 ± 22 for stage III, and 31 ± 10 for stage IV. Of the 32 cases with obstructive disease, the mean FEV1 was 69 ± 10 for stage 0 (*n* = 5), 53 ± 18 for stage II (*n* = 15), 55 ± 14 for stage III (*n* = 10), and 27 ± 6 for stage IV (*n* = 2). No subject with obstructive pattern had stage I disease.

**Table 4:** Demographics by Scadding stage

Stage	0	I	II	III	IV	*p* value
Number of subjects	70 (37.4%)	21 (11.2%)	47 (25.1%)	45 (24.1%)	4 (2.1%)	
Age at enrollment (years)	52.5 ± 12.2	49.0 ± 12.5	53.9 ± 13.2	54.5 ± 12.7	53.5 ± 9.7	0.55
Age at diagnosis (years)	47.1 ± 12.3	43.2 ± 11.7	45.9 ± 13.9	45.0 ± 11.6	41.5 ± 4.2	0.67
BMI (kg/m^2^)	31.5 ± 7.9	30.3 ± 5.0	31.2 ± 5.2	31.6 ± 8.5	29.3 ± 4.1	0.94
Sex						
Male	30 (16.0)	12 (6.4)	31 (16.6)	26 (13.9)	4 (2.1)	0.04
Female	40 (21.4)	9 (4.8)	16 (8.6)	19 (10.2)	0 (0.0)	
Race						
White	52 (27.8)	12 (6.4)	38 (20.3)	29 (15.5)	1 (0.5)	0.04
Black	9 (4.8)	3 (1.6)	7 (3.7)	11 (5.9)	2 (1.1)	
Asian	3 (1.6)	0 (0.0)	0 (0.0)	0 (0.0)	0 (0.0)	
American Indian or Alaska Native	1 (0.5)	0 (0.0)	0 (0.0)	0 (0.0)	0 (0.0)	
Choose not to answer	5 (2.7)	6 (3.2)	2 (1.1)	5 (2.7)	1 (0.5)	
Smoking status						
Current	6 (3.2)	2 (1.1)	3 (1.6)	3 (1.6)	1 (0.5)	0.27
Former	28 (15.0)	3 (1.6)	18 (9.6)	20 (10.7)	2 (1.1)	
Never	36 (19.3)	16 (8.6)	26 (13.9)	22 (11.8)	1 (0.5)

### Treatment groups

The majority (71.1%) of subjects reported treatment for at least 1 month for sarcoidosis. Systemic anti-inflammatory agents for greater than three months were needed in 123 (65.8%) cases, and only 22 (11.8%) were off therapy at one-year anniversary of start treatment (Table 5). Prednisone was the first agent for a majority (70.1%) of the cases, and methotrexate and Imuran were used in 59 (31.6%) and 9 (4.8%) cases respectively. Fourteen cases received infliximab with a preferred dose of 5 mg/kg for induction (0, 2 and 4 weeks) and a maintenance dose given every 4 weeks. Solumedrol 1 g once a month was used in cases with cardiac disease and when there were adverse effects or intolerance of conventional oral or biologic agents. 31.0% of subjects were treated with only one anti-inflammatory agent, while 23.5% were treated with two and 10.7% with three anti-inflammatory agents (Table 6) for management of sarcoidosis over the duration of illness.

**Table 5:** Treatment groups by duration and agents

Treatment Groups	*n*	%
Treated < 1 month or never	54	28.9
Treatment ≥1 month	133	71.1
Treatment > 3 months	123	65.8
Off treatment ≥12 months	22	11.8
Prednisone	131	70.1
Methylprednisolone	13	7.0
Methotrexate	59	31.6
Azathioprine	9	4.8
Mycophenolic acid	13	7.0
Hydroxychloroquine	16	8.6
Infliximab	14	7.5

**Table 6:** Number of immunosuppression agents used in each subject

Number of agents used	*n* (%)
0	54 (28.9%)
1	58 (31.0%)
2	44 (23.5%)
3	20 (10.7%)
4	7 (3.7%)
5	4 (2.1%)

### Clinical phenotypes per GRADS

Clinical phenotypes were determined using the GRADS study criteria. Cases with remitting disease, i.e., subjects who were never treated or were off treatment for greater than 12 months, were the largest group (*n* = 45 [24.1%]) in this cohort (Table 7). Of those classified into Group 8, five subjects had multiorgan involvement while four subjects had cardiac involvement. We then evaluated the proportion of cases with parenchymal disease based on GRADS phenotyping. Forty-four (23.5%) subjects were classified into Group 3 Stage II-III treated, with 23 (12.3%) subjects classified into Group 4 Stage II-III untreated. Meanwhile, only 1.1 and 0.5% of subjects were classified into Group 5 Stage IV treated and Group 6 Stage IV untreated, respectively. A substantial number of cases were not classifiable based on GRADS phenotyping (*n* = 37 [19.8%]), including 4.3% with Stage I who were treated, 14.4% with Stage 0 treated, and 1.1% with Stage 0 untreated. Some of the cases who met criteria for Group 1 (multiorgan disease) or Group 9 (cardiac defining therapy) were not included for the respective groups if they met criteria for remitting disease. For instance, while 25 subjects had multiorgan involvement, only 16 of them were classified into Group 1 Multiorgan since six of them were classified into Group 8 Remitting and three of them were classified into Group 9 Cardiac defining therapy. Likewise, while 20 subjects had cardiac involvement, only seven of them who were treated for cardiac involvement and not remitting were classified into Group 9 Cardiac defining therapy.

**Table 7:** Clinical phenotype study groups per GRADS

Clinical Phenotype Study Groups	*n*	%
Group 1: Multiorgan	16	8.6
Group 2: Nonacute, Stage I, untreated	8	4.3
Group 3: Stage II-III, treated	44	23.5
Group 4: Stage II-III, untreated	23	12.3
Group 5: Stage IV, treated	2	1.1
Group 6: Stage IV, untreated	1	0.5
Group 7: Acute sarcoidosis	4	2.1
Group 8: Remitting	45	24.1
Group 9: Cardiac defining therapy	7	3.7
Unclassifiable per GRADS	37	19.8
Stage 1, treated	8	4.3
Stage 0, treated	27	14.4
Stage 0, untreated	2	1.1

### Laboratory values

The sarcoidosis relevant laboratory studies for this cohort are shown in Table 8. A higher WBC count (*p* = 0.003), and lower albumin (*p* = 0.04) was seen in cases who were treated. White cases had lower IgG (*p* = 0.01), higher albumin (*p* = 0.01), and lower total protein levels (*p* = 0.002) compared to blacks. No significant difference between treated and untreated groups, and between whites and blacks was found for ACE, percent lymphocytes, soluble IL2 receptor, CRP, total bilirubin, alkaline phosphatase, alanine aminotransferase, aspartate aminotransferase, calcium, parathyroid hormone, 25-OH Vitamin D, and 1,25 Di OH Vitamin D.

**Table 8:** Lab values between treated vs untreated, and whites and blacks

Characteristics	Total		Treated		Untreated			White		Black		
	n	mean ± SD	n	mean ± SD	n	mean ± SD	*p*-value	n	mean ± SD	n	mean ± SD	*p*-value
ACE (U/L)	124	43.6 ± 40.6	85	42.7 ± 43.1	39	45.7 ± 35.0	0.68	90	43.8 ± 39.8	19	50.9 ± 51.7	0.58
WBC (10^9^/L)	176	6.7 ± 2.6	128	7.0 ± 2.7	48	5.9 ± 1.9	0.003	127	6.7 ± 2.4	29	6.1 ± 2.2	0.17
Lymphocytes (%)	169	21.4 ± 20.1	123	20.8 ± 22.9	46	23.2 ± 8.9	0.32	120	21.2 ± 22.7	29	23.5 ± 11.1	0.43
sIL-2R (unit/mL)	47	599.8 ± 306.9	33	580.5 ± 317.4	14	645.5 ± 286.5	0.50	32	600.9 ± 273.1	6	597.8 ± 452.1	0.99
CRP (mg/L)	96	12.8 ± 29.0	65	13.3 ± 31.2	31	11.8 ± 24.1	0.80	64	8.3 ± 13.6	17	28.8 ± 59.1	0.17
IgG (mg/dL)	91	1171.9 ± 379.8	60	1136.5 ± 362.9	31	1240.6 ± 407.7	0.22	60	1070.8 ± 277.4	16	1492.3 ± 555.5	0.01
Albumin (g/dL)	172	3.8 ± 0.4	123	3.8 ± 0.4	49	3.9 ± 0.3	0.04	122	3.9 ± 0.4	28	3.7 ± 0.3	0.01
Total protein (g/dL)	172	7.6 ± 0.6	123	7.6 ± 0.6	49	7.7 ± 0.5	0.09	122	7.5 ± 0.5	28	7.9 ± 0.6	0.002
Total bilirubin (mg/dL)	172	0.6 ± 0.3	123	0.6 ± 0.4	49	0.6 ± 0.3	0.86	122	0.6 ± 0.3	28	0.6 ± 0.4	0.65
ALP (U/L)	172	91.1 ± 44.7	123	93.6 ± 44.4	49	84.7 ± 45.3	0.25	122	86.9 ± 39.5	28	112.3 ± 66.3	0.06
ALT (U/L)	172	35.8 ± 16.1	123	36.9 ± 17.1	49	33.0 ± 12.8	0.11	122	36.4 ± 15.7	28	32.8 ± 18.0	0.33
AST (U/L)	172	24.4 ± 11.2	123	25.2 ± 12.2	49	22.4 ± 7.8	0.08	122	23.8 ± 9.4	28	24.7 ± 13.0	0.72
Calcium (mg/dL)	177	9.1 ± 0.6	128	9.1 ± 0.6	49	9.2 ± 0.5	0.18	125	9.1 ± 0.6	30	9.2 ± 0.6	0.39
PTH (pg/mL)	46	53.5 ± 38.5	32	58.5 ± 44.2	14	42.1 ± 16.3	0.07	30	55.7 ± 35.6	8	48.9 ± 62.1	0.77
25, OH Vit D (ug/L)	99	29.5 ± 18.0	70	28.8 ± 19.2	29	31.1 ± 14.9	0.52	67	28.4 ± 16.2	15	31.7 ± 21.4	0.57
1, 25 DiOH Vitamin D (pg/mL)	88	53.1 ± 21.1	61	51.4 ± 21.8	27	56.9 ± 19.2	0.24	59	50.7 ± 19.4	12	59.0 ± 24.2	0.28

## Discussion

Our cohort has similarities and crucial differences compared to previously described cohorts (Table 9). This cohort is primarily composed of cases from upper Midwest and has a higher proportion of blacks and lower proportion of whites compared to the racial distribution of Minnesota residents. With respect to the ACCESS cohort, the age at diagnosis in our cohort was similar, but we observed a higher proportion of males and a lower proportion of black subjects in our cohort. We also observed a higher proportion of extra-thoracic lymph node, eye, liver, spleen, and bone-joint involvement. Other cohorts also reported similarities and differences compared to the ACCESS cohort. A cohort of 166 sarcoidosis cases in Israel was older [17]. Similar to ACCESS cases, more patients in the Israeli cohort were females, and the majority of patients had lung involvement. However, fewer sarcoidosis cases in this cohort had extra thoracic lymph node, liver, CNS, and joints involvement. A small cohort of 21 cases form Mexico was different than the ACCESS cohort with a lower proportion of women and lower median age [16]. This group had more frequent skin and bone marrow involvement and less frequent pulmonary involvement. In contrast, a cohort from the University of California [24] found a higher proportion of cases with neurologic involvement, and another cohort in Olmsted County in Minnesota [25] had fewer cases with eye involvement, but more bone and joint disease. In our cohort, in addition to higher proportion of extra-thoracic lymph node, eye, liver, spleen, and bone-joint involvement, cardiac sarcoidosis, a high-risk manifestation of sarcoidosis, was also more frequent. Given the interaction between the environmental factors and genetics in the pathogenesis of sarcoidosis, regional variations in disease phenotypes may be due to differences in the genetics of the population and/or the nature of the exposure(s) triggering the disease. Because our cohort is drawn from a referral center for sarcoidosis, there may be enrichment for more complicated and advanced cases.

**Table 9:** Demographics and organ involvement characteristics of various cohorts

Characteristics	UMN (*n* = 187)	ACCESS (*n* = 736)	Israel (*n* = 166)	Mexico City (*n* = 21)	UCSF (*n* = 126)	Olmsted County (*n* = NA)
Mean age ± SD (years)	45.8 ± 12.4^a^	(46% < 40 yr old, 54% ≥ 40 yr old)^a^	62 ± 14	NA	50 ± 12^b^	NA
Median age (years)	45 (22–75)^a^	42.1 (18–83)^a^	63 (26–94)	31 (18–72)^a^	51 (30–76)^b^	NA
Male, *n* (%)	103 (55.1)	268 (36.4)	54 (32.5)	10 (47.6)	49 (39)	NA
Female, *n* (%)	84 (44.9)	468 (63.6)	112 (67.5)	11 (52.4)	77 (61)	NA
White, *n* (%)	132 (70.6)	393 (53.4)	NA	NA	97 (77)	NA
Black, *n* (%)	32 (17.1)	325 (44.2)	NA	NA	18 (14)	NA
Other, *n* (%)	23 (12.3)	18 (2.4)	Jew: 111 (67) Arab: 51 (31) Ethiopian 4 (2)	NA	11 (9)	NA
Lungs, *n* (%)	184 (98.4)	699 (95.0)	151 (91.0)	14 (66.6)	NA	97%
Skin^c^, *n* (%)	27 (14.4)	178 (24.2)	5 (3.0)	9 (42.8)	NA	18%
Non-thoracic lymph node, *n* (%)	64 (34.2)	112 (15.2)	5 (3.0)	4 (19.0)	NA	3%
Eye, *n* (%)	39 (20.9)	87 (11.8)	6 (3.6)	4 (19.0)	NA	7%
Liver, *n* (%)	33 (17.6)	85 (11.5)	6 (3.6)	4 (19)	NA	6%
Spleen, *n* (%)	39 (20.9)	49 (6.7)	6 (3.6)	0 (0.0)	NA	4%
Neurologic, *n* (%)	14 (7.5)	34 (4.6)	12 (7.2)	0 (0.0)	23 (18.3%)	3%
Parotid/salivary, *n* (%)	4 (2.1)	29 (3.9)	0 (0.0)	1 (4.7)	NA	NA
Bone marrow, *n* (%)	6 (3.2)	29 (3.9)	1 (0.6)	5 (23.8)	NA	NA
Calcium, *n* (%)	10 (5.3)	27 (3.7)	1 (0.6)	1 (4.7)	NA	NA
ENT, *n* (%)	4 (2.1)	22 (3.0)	0 (0.0)	1 (4.7)	NA	NA
Cardiac, *n* (%)	20 (10.7)	17 (2.3)	1 (0.6)	0 (0.0)	5 (4.0%)	1%
Renal, *n* (%)	2 (1.1)	5 (0.7)	4 (2.4)	0 (0.0)	NA	3%
Bone/joint, *n* (%)	18 (9.6)	4 (0.5)	6 (3.6)	0 (0.0)	NA	12%
Muscle, *n* (%)	2 (1.1)	3 (0.4)	0 (0.0)	0 (0.0)	NA	NA

*ENT* ear, nose, and throat, *UMN* University of Minnesota, *ACCESS* A Case Control Etiologic Study of Sarcoidosis, *UCSF* University of California San Francisco

^a^Age at diagnosis

^b^Age at visit

^c^Including erythema nodosum

Pulmonary sarcoidosis continues to result in significant morbidity and mortality with rising death rates [10], hospital admissions, and health care costs [2]. It is associated with significantly reduced health related quality of life and symptoms portending the need for treatment and for disease progression [26]. A large proportion of our cases were either stage II/III disease or stage 0 disease, and stage IV primarily fibrotic disease is less common. A substantial proportion of our cases had near normal spirometry and DLCO, and restrictive ventilatory defect was the most frequent finding on pulmonary function testing compared to obstructive and mixed patterns. A large proportion of subjects in our cohort required treatment with anti-inflammatory agents. In the ACCESS incident cohort, lung function was normal in many with FVC > 80% in 69%, yet ~ 47% cases had Scadding stage II/III disease while stage IV disease was present in only ~ 5%. In our cohort, the median and 25th percentile of FVC is 88% predicted and 72% predicted, findings similar to ACCESS. Similar results were also seen for FEV1, and obstructive changes were common with an FEV1/FVC ratio of < 70% in more than 20% of patients. An isolated gas exchange abnormality is not uncommon in sarcoidosis and thus an important clinical outcome. In our cohort, 14.0% had an abnormal DLCO and normal TLC, while 6.6% had abnormal DLCO and normal spirometry, and 17% demonstrated normal DLCO and abnormal spirometry. Thus, patients may demonstrate obstructive, restrictive, mixed patterns or an isolated DLCO. Researchers have commonly used spirometry, DLCO, and chest radiography, to track disease progression in studies [27, 28]. Lung volumes or formal cardiopulmonary exercise testing (CPET) is not routinely used nor is a standard of care in sarcoidosis clinical practice or research as it is more invasive, expensive and normal in many [29]. Patient-reported outcome measures and the six-minute walk test have been recommended in disease assessment [30]. A recent international Delphi study supported multi-faceted outcomes to assess disease severity for clinical and research purposes, including spirometry/DLCO, quality of life, assessment of progression as well as a biomarker [31]. These data support the importance of defining a comprehensive biomarker of disease progression.

A recent NHLBI workshop recognized cardiac involvement as a high-risk phenotype [12, 32]. The proportion of sarcoidosis cases with abnormal advanced cardiac imaging was substantially higher in our cohort than the ACCESS and other cohorts (Table 9), but less than the reported incidence in post mortem studies [33–35]. Similar rates of increased detection of cardiac sarcoidosis has also been reported by other investigators employing contemporary imaging studies [36–38]. The utility of these imaging modalities is now widely recognized and incorporated in the diagnostic criteria for cardiac sarcoidosis. The ACCESS criteria did not have advanced imaging criteria, and for the 2006 Japanese Ministry of Health and Welfare (JMHW) criteria, late gadolinium enhancement (LGE) on cardiac MRI was a minor criterion [39] and needed additional criteria to be fulfilled for a diagnosis of cardiac sarcoidosis. In contrast, in the current recommendations by Heart Rhythm Society (HRS), LGE on CMR is sufficient for an adequate clinical diagnosis of cardiac disease in the presence of histologic extracardiac evidence of sarcoidosis [40]. Similarly, for the WASOG organ assessment tool that we used for this cohort [18] and the Japanese Circulation Society Guidelines [41], LGE on cardiac MRI makes the likelihood of cardiac involvement at least probable. Similarly, patchy uptake on a dedicated cardiac PET scan has also been incorporated into the HRS and the WASOG criteria. A crucial element in establishing a diagnosis of cardiac sarcoidosis, even with histology proven extra-cardiac disease, is excluding other causes of LGE, such as coronary artery disease or other non-ischemic cardiomyopathies such as genetic arrhythmogenic cardiomyopathies which could also demonstrate increased myocardial uptake on FDG-PET imaging [42]. Challenges also remain regarding the ideal dietary preparation to suppress normal glucose uptake by the myocardium and avoid false positive FDG-PET. A diffuse uptake of FDG is suggestive of incomplete suppression of physiological glucose uptake in contrast to a patchy or patchy on diffuse uptake that may suggest myocardial inflammatory process [43, 44]. In the absence of a gold standard, as suggested by other groups [45], a multidisciplinary approach is likely to improve the accuracy of diagnosis. The long-term outcomes of cases with abnormal cardiac imaging, especially in cases with no or minimal symptoms and normal heart function, remains unknown.

Extra-pulmonary involvement is presumed to be more common in blacks with sarcoidosis. However despite being a cohort composing of predominantly white cases, our cohort had frequent multisystem disease (using the GRADS criteria and also the WASOG organ assessment tool). We observed more frequent involvement of the skin, extra thoracic lymph nodes, liver, spleen, neurologic, cardiac and bone and joint disease. Of these, neurologic, cardiac, and multisystem disease were considered high-risk manifestations of sarcoidosis due to higher morbidity and mortality. Bone and joint involvement is also a frequent cause of impaired quality of life and often requires systemic anti-inflammatory therapy. We included cases with Lofgren’s syndrome in this category due to our observation of significant morbidity during the acute presentation related to arthralgias, although most of these cases had improvement in symptoms and did not need chronic therapy. As the size of our cohort grows, it will be ideally suited for future studies of high-risk sarcoidosis phenotypes.

We also examined laboratory studies obtained at the clinic visits. Routine laboratory tests were performed for surveillance of drug-related side effects to assess the disease activity with biomarkers and for asymptomatic organ involvement [46]. We identified differences in several analytes in the treated vs. untreated groups and in blacks compared to whites. A higher leukocyte count in treated sarcoidosis cases could reflect an appropriate response to therapy including improved hematopoiesis [47]. Leukopenia as a manifestation of sarcoidosis could be due to bone marrow involvement [48, 49], hypersplenism [50], or lymphocyte redistribution. The T cell redistribution is reported to cause peripheral lymphopenia and is linked to worse severity of the sarcoidosis [51, 52]. We did not observe any difference in the lymphocyte count in the treated vs. untreated and blacks vs. whites. Our cohort is larger in size than the cohort where peripheral lymphopenia was observed and has a larger proportion of cases with lung involvement [52]. Other potential biomarkers of disease activity such as angiotensin converting enzyme [53, 54] and soluble IL-2R levels [55, 56] were also not different in treated vs untreated groups or in whites vs. blacks. The higher level of albumin observed in whites and a higher level of total protein in blacks has been reported previously [57] in these racial groups. Difference in protein fractional patterns could explain a higher IgG level. Various reasons including frequent infections or persistent chronic inflammation could account for the differences in albumin and immunoglobulin levels [58, 59]. Overall, these findings are in line with the findings of other reports that current biomarkers have limited utility for assessing disease activity in sarcoidosis, and highlight the urgent need to develop biomarkers for prognostication and measuring response to therapy [32]. Concurrent to the search for useful serum biomarkers for disease activity, methods for incorporating comprehensive data including other assessments of disease activity, extent of organ dysfunction, and patient reported outcomes, are needed to improve disease severity assessment, prognosis assessment, and guidelines for treatment.

We observed a high proportion of cases that needed systemic anti-inflammatory therapy. Oral corticosteroids are the first-line agents with methotrexate and Imuran as the preferred steroid sparing agent. The dosing of TNF blockers used in sarcoidosis at our center is different than the typical dose for rheumatologic conditions with a maintenance dose of every four weeks (5 mg/kg) for infliximab, in line with the current experience-based recommendations [60].

Despite the retrospective nature of our study, this cohort description provides new insights into regional variability of sarcoidosis as it represents the clinical manifestation in residents of the upper Midwest. Our cohort is a mixed population of incident and prevalent cases and might be a more appropriate representation of sarcoid-related morbidity as opposed to a cohort of newly diagnosed cases. For assessment of pulmonary function testing, we used percent predicted values to assess lung function abnormalities. The other option of using percentile cutoff might be more appropriate, but we do not have this information on all the participants in our study. We also acknowledge that not all the cases in our cohort had all the data points that we presented. As our cohort size grows, we expect the missing values to have a lesser impact on the study findings.

## Conclusion

Among sarcoidosis cases in our predominantly white Minnesota population, we found a relatively high proportion of advanced and multisystem disease. Lofgren’s syndrome was not common in our cohort despite high percentage of presumed north-European ancestry in our population. Stage II/III pulmonary disease, cardiac disease, multisystem diseases and joint and bone diseases are more frequently detected in this cohort. The analysis of our cohort highlights the lack of utility of serum biomarkers and limitations of pulmonary function tests in assessing prognosis, need for treatment, and disease severity. The higher incidence of cardiac disease, compared to previous cohorts, suggests that cardiac involvement may be under-recognized and highlights the importance of incorporating advanced imaging and a multidisciplinary approach into the evaluation of patients suspected of cardiac involvement. Our cohort is well suited to investigate high-risk sarcoidosis phenotypes, and studying regional variations in disease phenotypes may shed light on disease mechanisms. Incorporating patient reported outcomes in routine care could provide additional insights into indications and outcomes of treatment.

## Data Availability

The datasets generated during and/or analyzed during the current study are not publicly available due to HIPPA regulations and are available from the corresponding author on reasonable request.

## Abbreviations

ACE: Angiotensin converting enzyme
ACCESS: A Case Control Etiologic Study of Sarcoidosis
ATS: American Thoracic Society
CBD: Chronic beryllium disease
CPET: Cardiac -positron emission tomography
CVID: Common variable immunoglobulin deficiency
DLCO: Diffusing capacity of the lungs for carbon monoxide
FDG: Fluorodeoxyglucose
FEV1: Forced expiratory volume in 1 s
FVC: Forced vital capacity
GRADS: Genomic Research in Alpha-1 Antitrypsin Deficiency and Sarcoidosis
HRS: Heart Rhythm Society
JMHW: Japanese Ministry of Health and Welfare
LGE: Late gadolinium enhancement
NASH: Nonalcoholic steatohepatitis
sIL-2R: Soluble interleukin 2 receptor
TLC: Total lung capacity
UCSF: University of California San Francisco
UMN: University of Minnesota
WASOG: World Association of Sarcoidosis and Other Granulomatous Disorders
WBC: White blood cell
